# Mediation of modifiable risk factors in two multidomain dementia prevention trials

**DOI:** 10.1002/alz.14557

**Published:** 2025-01-27

**Authors:** Marieke P. Hoevenaar‐Blom, Jason Shourick, Jan Willem van Dalen, Willem A. van Gool, Sandrine Andrieu, Edo Richard, Nicola Coley, Eric Moll van Charante, the preDIVA study group, MAPT/IHU HealthAge Open Science study group

**Affiliations:** ^1^ Department of Public and Occupational Health Amsterdam UMC Location VUMC Amsterdam the Netherlands; ^2^ Department of General Practice Amsterdam UMC Location AMC Amsterdam the Netherlands; ^3^ Aging Research Team, Centre for Epidemiology and Research in Population Health (CERPOP), INSERM‐University of Toulouse UPS Toulouse France; ^4^ Department of Epidemiology and Public Health Toulouse University Hospital Toulouse France; ^5^ IHU HealthAge, Cité de la santé, place Lange Toulouse France; ^6^ Department of Neurology Amsterdam UMC, University of Amsterdam Amsterdam the Netherlands; ^7^ Department of Neurology Donders Centre for Brain Behaviour and Cognition Radboud University Medical Center Nijmegen the Netherlands

**Keywords:** dementia, mediation, multidomain intervention, prevention, risk factors, trial

## Abstract

**INTRODUCTION:**

We explored which dementia risk factors in two multidomain prevention trials mediate beneficial, neutral, or counteracting effects on dementia incidence.

**METHODS:**

We pooled data from the multidomain MAPT (Multidomain Alzheimer Preventive Trial; *n* = 1679, up to 5‐year follow‐up) and preDIVA trials (Prevention of Dementia by Intensive Vascular Care; *n* = 3526, up to 12‐year follow‐up) in adults aged 70+. We used multiple mediation analysis to quantify the role of 2‐year changes in body mass index, systolic blood pressure, total cholesterol, and physical activity in the intervention effects on dementia incidence. Mixed linear and Cox proportional hazard models were used to explore pathways.

**RESULTS:**

We observed no mediation of individual risk factors in the effect of the interventions on dementia incidence. The interventions slightly lowered only blood pressure, but this did not translate into an effect on dementia incidence.

**DISCUSSION:**

In older populations, multidomain interventions may not sufficiently affect dementia risk factors to lower dementia incidence, particularly in settings where cardiovascular risk factor management is well implemented.

**Highlights:**

- There is no mediating role of risk factor change in the effects of multidomain interventions on dementia incidence in two large dementia prevention randomized controlled trials.
- The lack of effect of the interventions on risk factors explains the absence of impact on dementia.
- Counteracting mediators do not explain the lack of effect of the interventions on dementia.
- A small effect of the interventions on blood pressure did not translate into a lower dementia incidence.

## 1 BACKGROUND

Observational studies suggest that up to 40% of dementia cases may be attributable to modifiable risk factors and that prevention strategies addressing multiple risk factors simultaneously are most likely to effectively modify dementia risk due to synergistic effects.^1^ In contrast to these promising associations, most randomized trials show no or only modest effects of multidomain interventions on cognitive decline and dementia.^2^, ^3^, ^4^

Investigating the mechanisms by which multidomain interventions do and do not impact dementia incidence is important to explain this contradiction. Quantifying the mediating effects of the risk factors might suggest which factors are most viable to target. Complex multidomain interventions might induce both beneficial changes in some risk factors and detrimental changes in others. An example is that lower blood pressure and body mass index (BMI) have been associated with higher dementia risk in older people.^5^, ^6^, ^7^ Therefore, we hypothesized that some risk factor change would have a beneficial mediating effect on dementia/cognition, whereas changes in other risk factors have a detrimental negative impact on dementia risk, thus reducing the overall intervention effect (i.e., counteracting mediators). Alternatively, as multidomain interventions often have a pragmatic design to make them feasible in a real‐life setting, the effect on the individual risk factors may be too small to translate into an effect on dementia.

While acknowledging potential counteracting mediating effects, we aimed to explain the lack of effects of multidomain interventions on dementia incidence and identify key risk factors to target in older people. Our objective was therefore to explore how changes in dementia risk factors mediate the impact of two large multidomain intervention trials^8^, ^9^ on dementia incidence.

## 2 METHODS

### 2.1 Multidomain Alzheimer Preventive Trial and Prevention of Dementia by Intensive Vascular Care trials

**FIGURE 1:**
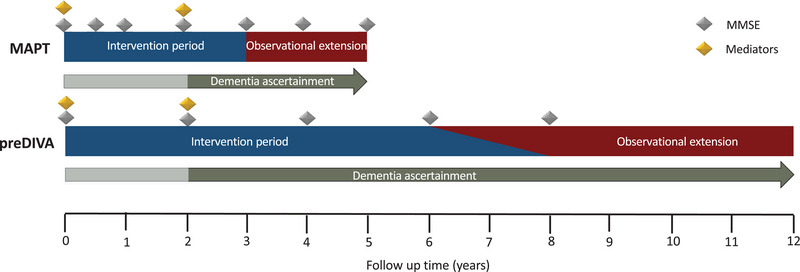
preDIVA and MAPT study timelines. MAPT, Multidomain Alzheimer Preventive Trial; MMSE, Mini‐Mental State Examination; preDIVA, Prevention of Dementia by Intensive Vascular Care.

The Multidomain Alzheimer Preventive Trial (MAPT) and Prevention of Dementia by Intensive Vascular Care (preDIVA) trials targeted lifestyle and cardiovascular risk factors for the prevention of cognitive decline and/or dementia in > 5200 individuals aged ≥ 70. The interventions lasted for 3 years (MAPT) and 6 to 8 years (preDIVA) and had an observational follow‐up duration of 2 years (MAPT) and 4 to 6 years (preDIVA) during which the intervention was not actively continued, but information on dementia was collected (Figure 1). In both studies, the interventions had no effects on cognitive decline nor on dementia incidence—though it should be noted that MAPT was not powered for effects on dementia incidence.^8^, ^9^, ^10^

The MAPT trial included 1679 community‐dwelling individuals aged ≥ 70 with subjective memory complaints, and/or instrumental activity of daily living (IADL) limitation and/or slow walking speed. MAPT tested a multidomain intervention comprising group sessions involving cognitive training, physical activity, and nutritional advice, as well as a yearly preventive consultation assessing cardiovascular and other dementia risk factors. The control group received usual care. The primary outcome was a change in cognitive function. Effects of omega‐3 fatty acid supplementation were also tested (MAPT had a parallel‐groups design), but in this study, we compared individuals who received the multidomain intervention regardless of omega‐3 assignment. Each participant received 3 years of intervention between 2008 and 2014, followed by an optional 2‐year period of extended observational follow‐up (Figure 1).^8^

The preDIVA trial included 3526 community‐dwelling individuals aged 70 to 78 and tested a nurse‐led multidomain cardiovascular risk factor management intervention consisting of lifestyle advice and drug treatment when necessary. The control group received usual care from their general practitioner (GP), which included regular cardiovascular risk management. The primary outcomes were dementia and disability. The intervention ran from 2006 to 2015, lasted 6 to 8 years for each participant, and follow‐up was extended up to 12 years (Figure 1).^9^, ^10^

Both trials were approved by local ethical committees, performed in accordance with the ethical standards as laid down in the 1964 Declaration of Helsinki and its later amendments, and participants gave written informed consent. The trials were registered at ClinicalTrials.gov (MAPT: NCT00672685) or the ISRCTN registry (preDIVA: ISRCTN29711771).

### 2.2 Outcome ascertainment

The primary outcome for the current study is the time to all‐cause dementia incidence between the year 2 visit (as mediator is the change between baseline and the 2‐year visit) and the end of follow‐up of both trials. Secondary outcomes were Alzheimer's disease (AD) dementia and cognition as measured by Mini‐Mental State Examination (MMSE) after 4 years of follow‐up in both trials.

In MAPT, dementia was ascertained during study evaluations conducted in hospital memory centers both during the intervention period and the extended follow‐up.^8^ In preDIVA, information on dementia status was collected from multiple sources including follow‐up visits during the trial, a Telephone Interview for Cognitive Status (TICS) for the extended follow‐up, and hospital‐ and GPs’ electronic health records and the National Death Registry during the trial and extended follow up.^9^, ^10^, ^11^ For both trials, a blinded outcome adjudication committee evaluated all cases also on the type of dementia. More details on the outcome ascertainment are described by Andrieu et al.^8^ for the MAPT trial, by Moll van Charante et al.^9^ for the preDIVA trial, and by Hoevenaar‐Blom et al.^10^ for the extended follow‐up for preDIVA.

**RESEARCH IN CONTEXT**

1. **Systematic review**: We reviewed the literature using PubMed. Randomized trials show no or modest effects of multidomain interventions on dementia and cognitive decline. In contrast, observational studies suggest that up to 40% of dementia cases may be attributable to modifiable risk factors.
2. **Interpretation**: It is challenging to create sufficient contrast between study arms in terms of modifying risk factors through lifestyle interventions to impact dementia incidence in older populations. The current interventions had no effect on body mass index, total cholesterol, or physical activity, but had a small blood pressure‐lowering effect, which did not translate into reduced dementia incidence.
3. **Future directions**: Interventions may need to be more intensive, targeted at higher risk populations, and augmented by a whole‐system approach to exert more substantial effects and thereby help prevent at least part of the dementia cases if attributable to these risk factors.

Cognitive function was measured during follow‐up at regular intervals with the MMSE in both trials, with up to seven repeated measures. We used the measurement 4 years after randomization as a secondary outcome as this is the latest common measurement point in the two trials (Figure 1).

### 2.3 Mediator assessment

The measurement of the mediators is extensively described in Appendix A in supporting information. Change in risk factors was defined as the difference between baseline and the 2‐year visit in physical activity (continuous, hours spent on activity of at least moderate intensity), systolic blood pressure (continuous, mmHg), BMI (continuous, kg/m^2^), and total cholesterol (continuous, mmol/L). The timeframe is set at “change in the first 2 years” as most change is expected to occur within the first 2 years^12^ and to allow for sufficient follow‐up time for dementia—as a mediator should precede the outcome. In a sensitivity analysis, we explored the mediating effects of change in smoking in preDIVA only, as this variable was not available for MAPT.

### 2.4 Statistical analyses

#### 2.4.1 General

Before analysis, all data were checked for missing values and miscoding, and univariate analyses were performed to check the distribution of variables and to identify abnormalities/outliers. Based on this, physical activity of at least moderate intensity was maximized at 5 hours per day.

All analyses were performed in complete cases according to the “intention to treat” principle for all randomized participants who underwent baseline and 2‐year assessments and had available outcome data. Baseline characteristics are presented as mean (standard deviation [SD]) or median (interquartile range) for continuous variables and frequency (percentage) for categorical variables.

All analyses were performed in R version 4.2.1.

#### 2.4.2 Models

All analyses were adjusted for potential confounders: study, baseline age (continuous), sex (dichotomous), education level (two dummy indicators), diabetes (dichotomous), and cardiovascular history (dichotomous), as well as change in (the other) risk factors (continuous) where applicable. In preDIVA, participants were cluster randomized on a GP level and in MAPT, participants were individually randomized. Therefore, a random intercept was added to all models for the trial, health care center (HCC) within the trial, and for preDIVA GP practice within HCC (except for multiple mediation analysis [MMA]). In a sensitivity analysis, we re‐ran all analyses without taking clustering into account.

#### 2.4.3 Non‐mediation analysis to explore pathways

In separate models we assessed (1) the effect of the interventions on intermediates (i.e., risk factors), (2) the associations of the intermediates with outcomes, and (3) the overall intervention effect on the outcomes. To assess effects/associations without a time‐to‐event outcome, a linear mixed model was used (R package “LME4”). To assess effects/associations with a time‐to‐event outcome, a random effect Cox proportional hazard model was used (R‐package “coxme”). The proportional hazard assumptions were verified graphically and with Schoenfeld residuals (Appendix B in supporting information).

#### 2.4.4 MMA

We used MMA^13^ from the R package “mma” to quantify the mediating role of changes in BMI, systolic blood pressure, total cholesterol, and physical activity in the effect of the interventions on cognitive function and dementia (indirect effects) as well as any effect over and above what these factors explain (direct effects). The MMA package uses counterfactual simulation to estimate the mediation effects of bootstrapping. MMA was chosen because it allows multiple mediators to be entered into the same model as well as time‐to‐event outcomes and bootstrap‐based confidence intervals to be estimated. The mediators were forced into the model. In a sensitivity analysis, we assessed the influence of the non‐linearity of associations using multiple additive regression trees (MART) within MMA.

The R code for the main MMA analysis can be found in Appendix C in supporting information.

## 3 RESULTS

**FIGURE 2:**
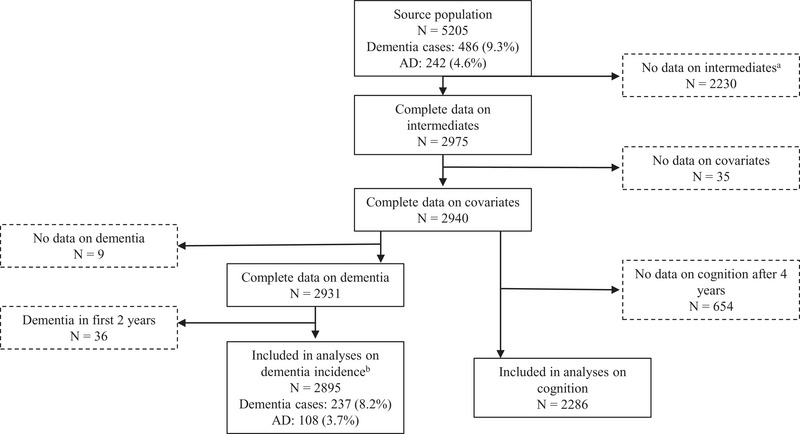
Flowchart of the study. ^a^No data on change in risk factors in the first 2 years. Mostly because participants did not attend the visit 2 years after randomization. ^b^Appendix D in supporting information shows the baseline characteristics of those included and excluded from the analyses. AD, Alzheimer's disease.

The pooled dataset included a total of 5205 individuals, of whom 486 (9.3%) developed dementia, including 242 cases of AD. Participants participated an average of 5.3 (SD 2.0) years in the trial and the average extended follow‐up after the trial was 2.0 (SD 2.1) years, totaling on average 7.3 (SD 3.7) years of follow‐up. Participants with missing data on mediators at the 2‐year visit (*n* = 2230), covariates (*n* = 35), and dementia incidence (*n* = 9) were excluded from the analyses. Also, people diagnosed with incident dementia in the first 2 years of follow‐up (*n* = 36) were excluded from the primary analyses as this preceded the measurement of the mediators, resulting in a dataset with 2805 participants (54% of the original population), 237 incident dementia cases, 108 incident AD cases (Figure 2). Participants that were not included in the analyses were slightly older, less educated, had higher blood pressure, were more likely to have a history of myocardial infarction (MI), and during follow‐up were more likely to be diagnosed with dementia and AD than those who were included (Appendix D in supporting information).

Compared to the MAPT study, at baseline, the preDIVA participants were less often female; less educated; had higher systolic blood pressure, BMI, and prevalence of diabetes, MI, and stroke; and lower total cholesterol levels (Table 1). For both studies combined, at baseline, there were no differences between the overall intervention and the control groups, except for slightly higher systolic blood pressure in the intervention group.

**TABLE 1:** Participants’ characteristics^a^.

	Trial		Randomisation group	
	MAPT	preDIVA	Control group	Intervention group
*N*	981	1914	1373	1522
Age, mean (SD)	75.1 (4.3)	74.4 (2.5)	74.6 (3.3)	74.6 (3.2)
Women, *N*(%)	635 (64.7%)	1069 (55.9%)	815 (59.4)	889 (58.4)
Level of education, *N*(%)				
Low	42 (4.3%)	444 (23.2%)	214 (15.6)	272 (17.9)
Medium	419 (50.1%)	1096 (57.3%)	758 (55.2)	829 (54.5)
High	448 (45.7%)	374 (19.5%)	401 (29.2)	421 (27.7)
Cognition (MMSE), median [IQR]	29 [27–29]	29 [27–29]	29 [28–29]	29 [27–29]
Systolic blood pressure (mmHg), mean (SD)^†^	140.3 (19.4)	155.2 (21.1)	149.1 (21.5)	151.1 (21.9)
Total cholesterol (mmol/L), mean (SD)	5.7 (1.1)	5.2 (1.1)	5.4 (1.1)	5.4 (1.1)
BMI (kg/m^2^), mean (SD)	26.0 (4.0)	27.5 (4.2)	27.0 (4.1)	27.1 (4.2)
Physical activity^b^ (hr/wk), mean (SD)	5.7 (5.8)	8.6 (9.6)	7.5 (8.5)	7.8 (8.7)
Diabetes, *N*(%)	74 (7.5%)	371 (19.4)	201 (14.6)	244 (16.0)
MI, *N*(%)	100 (10.2%)	537 (28.3)	289 (21.2)	348 (23.0)
Stroke, *N*(%)	52 (5.3%)	176 (9.3)	109 (8.0)	119 (7.9)
Dementia incidence, *N*(%)	27 (2.8%)	210 (11.0)	107 (7.8%)	130 (8.5%)
AD incidence, *N*(%)	23 (2.3%)	85 (4.5%)	47 (3.4%)	61 (4.0%)

Abbreviations: AD, Alzheimer's disease; BMI, body mass index; hr/wk, hours per week; IQR, interquartile range; MAPT, Multidomain Alzheimer Preventive Trial; MI, myocardial infarction; MMSE, Mini‐Mental State Examination; preDIVA, Prevention of Dementia by Intensive Vascular Care; SD, standard deviation.

^a^Of those included in analyses with dementia outcomes. Baseline characteristics unless otherwise indicated.

^b^ Of moderate or vigorous intensity.

^†^ *P* < 0.05 for control versus intervention.

**FIGURE 3:**
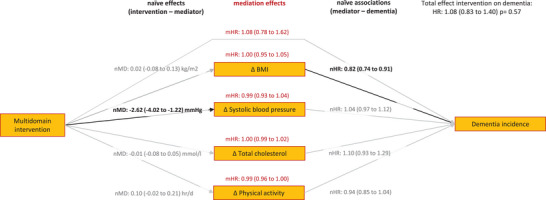
The mediating role of change in dementia risk factors in the effect of multidomain interventions on dementia incidence. All results are adjusted for baseline levels of mediators, age, sex, education level, diabetes, and cardiovascular diseases. Naïve models had a random intercept for trial/center/GP practice and the mediation model was adjusted for trial. This figure shows a small effect of the interventions on blood pressure (nMD: −2.62 mmHg), which did not translate into lower dementia incidence. No effects on the other mediators. This resulted in no mediation by the risk factors (all mHRs being close to 1.00). BMI, body mass index; GP, general practitioner; HR, hazard ratio; mHR, hazard ratio in mediation model; nHR, naïve hazard ratio of dementia incidence starting 2 years after randomization—for each unit change in the risk factor (per 10 mmHg for blood pressure); nMD, naïve mean difference in change of the risk factor in the first 2 years of the trial between the intervention and control group.

Figure 3 gives an overview of the results of all mediation analyses. There were no indirect effects of changes in systolic blood pressure, BMI, total cholesterol, or physical activity on the effect of the interventions on dementia incidence. In other words, there was no mediation of individual risk factors in the effect of the interventions on dementia incidence.

There was no effect of the interventions on dementia incidence (Table 2). The “naïve” non‐mediation analyses showed an effect of the interventions on change in blood pressure in the first 2 years (mean difference [MD]: −2.62 mmHg; [95% confidence interval (CI): −4.02 to −1.22]; Table 2), but a change in blood pressure was not associated with dementia incidence (Table 3). There was no effect of the interventions on change in BMI, total cholesterol, or physical activity (Table 2), though an increase in BMI was associated with lower dementia incidence (MD: 0.82 [95% CI: 0.74 to 0.91]; Table 3). The proportional hazard assumption did not hold for the association between total cholesterol and dementia incidence: Schoenfeld residual goodness‐of‐fit test *P* value: 0.008. There was no association in the first 8 years after the start of the interventions, and after 8 years the hazard ratio (HR) was 1.31 (1.00 to 1.72). When only including dementia cases after 8 years of follow‐up, there was still no indirect effect of changes in total cholesterol on the effect of the interventions on dementia incidence (HR: 1.00 [0.95 to 1.17]).

**TABLE 2:** Effects of multidomain interventions on intermediates and outcomes.

	*N*	Crude model	Adjusted model
**Effect of interventions on intermediates** ^a^			
BMI change (MD, kg/m^2^)	2895	0.01 (−0.09 to 0.11)	0.02 (−0.08 to 0.13)
Blood pressure change (MD, mmHg)	2895	−3.62 (−5.29 to −1.94)	−2.62 (−4.02 to −1.22)
Total cholesterol change (MD, mmol/L)	2895	−0.02 (−0.08 to 0.05)	−0.01 (−0.08 to 0.05)
Physical activity change (MD, quintile of time moderate/vigorous activity)	2895	0.10 (−0.03 to 0.22)	0.10 (−0.02 to 0.21)
**Effects of interventions on outcomes**			
Dementia incidence (HR)	2895	1.05 (0.81 to 1.36)	1.09 (0.84 to 1.41)
Cognition (MD, MMSE)^b^	2286	0.03 (−0.13 to 0.17)	0.05 (−0.09 to 0.19)

*Note*: All models have a random intercept for trial/center/GP practice. Adjusted model: adjusted for baseline mediator, age, sex, education level, diabetes, cardiovascular disease, change in (the other) intermediate factors. Results of stepwise adjustment can be found in Appendix F in supporting information. Effects on were analyzed in a dataset with complete data on dementia, and results in a dataset with complete data on cognition were similar.

Abbreviations: BMI, body mass index; GP, general practitioner; HR, hazard ratio; MD, the mean difference between the intervention and control group; MMSE, Mini‐Mental State Examination at year 4.

^a^ Change between baseline and 2 years of follow‐up.

^b^ MMSE score at 4 years of follow‐up, all models additionally adjusted for baseline MMSE.

**TABLE 3:** Associations of intermediates with dementia incidence (HR) and cognition (MD).

	*N*	Crude model	Adjusted model
**Dementia incidence (HR [95% CI])**			
BMI change (per kg/m^2^)	2895	0.83 (0.76 to 0.92)	0.82 (0.74 to 0.91)
Blood pressure change (per 10 mmHg)	2895	1.02 (0.96 to 1.09)	1.04 (0.97 to 1.12)
Total cholesterol change (per mmol/L)	2895	1.09 (0.94 to 1.26)	1.10 (0.93 to 1.29)
Physical activity change^a^	2895	0.93 (0.85 to 1.01)	0.94 (0.85 to 1.04)
**Cognition** ^b^ **(MMSE, MD [95% CI])**			
BMI change (per kg/m^2^)	2286	0.07 (0.02 to 0.12)	0.06 (0.01 to 0.11)
Blood pressure change (per 10 mmHg)	2286	0.03 (0.00 to 0.07)	0.02 (−0.02 to 0.06)
Total cholesterol change (per mmol/L)	2286	0.04 (−0.04 to 0.11)	0.02 (−0.06 to 0.10)
Physical activity change^a^	2286	−0.00 (−0.05 to 0.04)	0.02 (−0.03 to 0.07)

*Note*: All models have a random intercept for trial/center/GP practice. Adjusted model: adjusted for baseline mediator, age, sex, education level, diabetes, cardiovascular disease, and change in (the other) intermediate factors. Stepwise adjustment can be found in Appendix F in supporting information.

Abbreviations: BMI, body mass index; CI, confidence interval; GP, general practitioner; HR, hazard ratio; MD, the mean difference between the intervention and control group; MMSE, Mini‐Mental State Examination at year 4.

^a^ Per quintile of time moderate/vigorous activity.

^b^ MMSE score at 4 years of follow‐up, all models additionally adjusted for baseline MMSE.

Results were very similar when not taking clustering into account and the non‐linear approach (MART) did not change the results for dementia and further attenuated effects for cognition (Appendix E in supporting information). Stepwise adjustments showed that none of the potential confounders had an effect on the results (Appendix F in supporting information).

The results were similar for the secondary outcomes of AD (Appendix G in supporting information) and cognition outcomes after 4 years (Tables 2, 3, 4).

**TABLE 4:** The mediating role of change in dementia risk factors in the effect of multidomain interventions on dementia incidence and cognition.

	Beta (95% CI)	HR (95% CI)
**Dementia incidence**		
Total effect	0.07 (−0.29 to 0.45)	1.07 (0.75 to 1.57)
Direct effect	0.09 (−0.25 to 0.48)	1.08 (0.78 to 1.62)
Indirect effect	−0.02 (−0.10 to 0.04)	0.98 (0.90 to 1.04)
Via BMI change	−0.00 (−0.05 to 0.05)	1.00 (0.95 to 1.05)
Via blood pressure change	−0.01 (−0.07 to 0.04)	0.99 (0.93 to 1.04)
Via cholesterol change	0.00 (−0.01 to 0.02)	1.00 (0.99 to 1.02)
Via physical activity change	−0.01 (−0.04 to 0.00)	0.99 (0.96 to 1.00)
**Cognition (MMSE at year 4)**		
Total effect	0.03 (−0.12 to 0.16)	n.a.
Direct effect	0.04 (−0.10 to 0.19)	n.a.
Indirect effect	−0.01 (−0.04 to 0.01)	n.a.
Via BMI change	0.00 (−0.00 to 0.01)	n.a.
Via blood pressure change	−0.01 (−0.04 to 0.00)	n.a.
Via cholesterol change	−0.00 (−0.01 to 0.00)	n.a.
Via physical activity change	0.00 (−0.01 to 0.01)	n.a.

*Note*: Beta: for dementia absolute effects are mean log(HR)s of 500 bootstrap samples with BCa bootstrap 95% confidence intervals between parentheses. For cognition, absolute effects are regression coefficients. All models are adjusted for trial, baseline mediator level, age, sex and education level, diabetes, and cardiovascular disease.

Abbreviations: BCa, bias‐corrected and accelerated; BMI, body mass index; CI, confidence interval; HR, hazard ratio; MMSE, Mini‐Mental State Examination.

## 4 DISCUSSION

The mediation analyses show that, in this population of community‐dwelling individuals aged ≥ 70, there were no counteracting mediators in the effect of two pragmatic multidomain interventions on dementia incidence. The interventions had no effect on 2‐year changes in BMI, total cholesterol, or physical activity, but had a small effect on 2‐year changes in blood pressure, which did not translate into an effect on dementia incidence. The same results were observed for the cognitive decline and AD incidence outcomes.

The modest overall pooled effect of our lifestyle interventions is comparable to that of similar interventions aimed at lowering risk factors in a community‐dwelling setting. In a meta‐analysis of 94 randomized controlled trials (*N* = 52 174), behavioral counseling interventions were associated with small reductions in continuous measures of blood pressure (MD: −1.8 mmHg [95% CI:−2.5 to −1.1]), total cholesterol levels (MD:−0.09 mmol/L [95% CI:−0.12 to −0.4]), and BMI (MD: −0.4 kg/m^2^ [95% CI:−0.7 to −0.3]) at 12 to 24 months follow‐up.^14^ The impact of the interventions may have been small due to their pragmatic nature.

The effect of the interventions on blood pressure in the first 2 years did not translate into a lower dementia incidence, despite the Syst‐Eur trial showing that lowering blood pressure by 7.0 mmHg in older individuals could reduce dementia risk (HR: 0.38 [95% CI: 0.23 to 0.64]).^15^, ^16^ The Systolic Blood Pressure Intervention Trial Memory and Cognition in Decreased Hypertension (SPRINT MIND) trial had—with an effect of 13.3 mmHg on systolic blood pressure—an HR of 0.83 (95% CI: 0.67–1.04) for probable dementia incidence, despite early study determination.^15^ We hypothesize that the lack of translation into an effect on dementia in our studies may be due to the relatively small effect on systolic blood pressure of −2.6 mmHg we observed, compared to 7.0 mmHg in the Syst‐Eur trial and 13.3 mmHg in the SPRINT MIND trial. ^15^, ^16^

The inverse association of BMI with dementia incidence we observed is consistent with literature^5^ and is likely due to reverse causality as people—on average—tend to lose weight before the dementia diagnosis.^6^

Our study has some limitations. First, our interventions were of modest intensity and we cannot exclude that a more intensive intervention would have yielded a larger effect. Also, the intervention success is likely dependent on the degree to which our participants adhered to the lifestyle interventions.^17^ In preDIVA, 81% of the participants were adherent to the multidomain intervention (> two multidomain sessions per year) and in MAPT, 54% of participants were adherent (≥ 75% of the multidomain sessions).^8^, ^9^ Second, our outcomes were a clinical diagnosis of (AD) dementia and cognition based on MMSE. For AD we had only 108 cases, limiting the power of the analyses. Also, the distinction between vascular and AD‐type dementia is not so clear in old age dementia considering the overlap in post mortem brain autopsy studies as well as in clinical expression.^18^ MMSE has a strong ceiling effect in a population without cognitive complaints and is not a stand‐alone instrument for assessment of cognition as it is intended for first screening.^19^ Perhaps with detailed neuropsychological testing (only available in MAPT), we might have been able to detect more subtle treatment effects. Third, to allow for a pooled analysis of the two trials, only mediators that were measured in both trials could be incorporated in the analyses. A measure of diet or smoking, for instance, would have been of additional value. With regard to smoking, we checked the mediation effect in preDIVA and found it not to be a mediator, mainly because only 3% of participants changed their smoking status in the first 2 years (Appendix H in supporting information). Fourth, of the full study population of 5205 persons, 2805 were incorporated in the analyses (54%); for the other participants one or more variables were missing—mostly because they did not attend the 2‐year visit and thus had missing values on change in risk factors in the first 2 years (Figure 1). Also, in MAPT, 41.5% of participants did not participate in the extended observational follow‐up period, resulting in no measurement of cognition and dementia incidence during this period. Using multiple imputation in the non‐mediation analyses did not change the results (Appendix I in supporting information). However, it must be noted that missingness is likely not completely at random, as those with (early) cognitive decline and a poorer health status may be more likely to not attend follow‐up assessments. Concordantly, participants that could not be included had a slightly higher age, less education, higher blood pressure, more often a history of MI, and more often incident dementia during follow‐up than those that could be included. It suggests that those who could have benefited most from the interventions, due to their increased risk profile, were the least likely to complete them as previously shown by Coley et al.^17^ This underscores the importance of complementing individual behavior change efforts with approaches targeting environmental or policy‐level changes. Fifth, clustering within HCCs could not be taken into account in MMA. This is not ideal from a methodological point of view, though it is unlikely to have influenced the results as the addition of random effects in the non‐mediation analyses did not change the results (Appendix F). Sixth, we cannot rule out the possibility that multidomain interventions targeting younger individuals might have an effect on dementia. In the preDIVA and MAPT trials, all participants were ≥ 70 years old, an age range in which dementia is more likely to occur within the follow‐up period. However, for many of the targeted risk factors, the strongest associations with dementia risk are observed in middle age.^1^ Furthermore, these associations may reverse in older age, likely due to reverse causality.^6^, ^7^ Therefore, our results cannot be generalized to younger populations nor to racial nor ethnic minorities or those from other geographic regions. Seventh, measurement error—particularly in physical activity—may have introduced random error, leading to bias toward the null. Finally, for ethical reasons, control participants were also advised to contact their GP when high risk factors were observed at the baseline measurements—resulting in lower contrast with the intervention group.

The main strength of our study lies in the use of data from two large randomized controlled trials, with extended follow‐up for dementia incidence ascertainment,^8^, ^10^ resulting in a robust sample size of > 5200 participants, with nearly 500 dementia cases, and a follow‐up duration of up to 12 years. Also, the studies were performed in both France and the Netherlands, enhancing the overall generalizability of the results.

The frequently cited estimate that “40% of dementia cases are attributable to modifiable risk factors”^1^ is often interpreted to mean that 40% of dementia can be prevented by addressing these risk factors. This assumption relies on the belief that the associations are causal and that all risk factors can be permanently eliminated in all individuals. This stands in stark contrast to the modest effects achieved by pragmatic lifestyle interventions, including ours. To exert more substantial effects, interventions aimed at individuals may need to be supplemented by a whole‐system approach—for potentially preventing at least part of the dementia cases if they are indeed attributable to these risk factors.^1^, ^20^, ^21^

## 5 CONCLUSION

In pooled analyses of two large multidomain prevention trials in people ≥ 70 years old, we observed no mediation of individual risk factors in the effect of the interventions on dementia incidence. There was a small effect on systolic blood pressure but no effect on total cholesterol, BMI, or physical activity. The small effect of the interventions on blood pressure did not translate into an effect on dementia risk. Potentially, pragmatic interventions are not robust enough to sufficiently influence risk factors to prevent dementia—particularly against the backdrop of a high level of standard cardiovascular risk management.

## CONFLICT OF INTEREST STATEMENT

Dr. Andrieu reports grants from Occitania Region (No 1901175) and European Regional Development Funds (MP0022856), has received payment/honoraria from Roche, and has served as a consultant for Biogen with personal compensation. The other authors report no conflicts of interest. Author disclosures are available in the supporting information.

## CONSENT STATEMENT

All human subjects provided informed consent.

## CLINICAL TRIAL REGISTRATION NUMBERS

MAPT: clinicaltrials.gov, NCT00672685

PreDIVA: ISRCTN registry, ISRCTN29711771

## Supporting information



Supporting Information

Supporting Information

## Data Availability

Individual de‐identified participant data from the MAPT and preDIVA trials are available with immediate effect to academic researchers, following approval of a methodologically sound research proposal by the MAPT/preDIVA data sharing committee and signature of a data access agreement. Enquiries or proposals for MAPT should be directed to nicola.coley@inserm.fr and guyonnet.s@chu‐toulouse.fr and for preDIVA to m.p.hoevenaarblom@amsterdamumc.nl.

## COLLABORATORS

### PreDIVA

The members of the preDIVA group are: Eric P. Moll van Charante, Edo Richard, Lisa S. Eurelings, Jan‐Willem van Dalen, Suzanne A. Ligthart, Emma F. van Bussel, Marieke P. Hoevenaar‐Blom, Marinus Vermeulen, Willem A. van Gool (Amsterdam Medical Centre, Amsterdam, the Netherlands). We are indebted to all practice nurses delivering the intervention and all general practitioners involved in the care for the participants, including the “Zorggroep Almere.” We thank our project manager C.E. Miedema for her role in coordinating the trial. We acknowledge the efforts of the interim committee members (Dr. A. de Craen†, Prof. N. de Wit and Prof. J. Stam). We thank the members of the independent outcome adjudication committee.

## MAPT/IHU HealthAge Open Science Group

MAPT Study Group—Principal investigator: Bruno Vellas (Toulouse); Coordination: Sophie Guyonnet; Project leader: Isabelle Carrié; CRA: Lauréane Brigitte; Investigators: Catherine Faisant, Françoise Lala, Julien Delrieu, Hélène Villars; Psychologists: Emeline Combrouze, Carole Badufle, Audrey Zueras; Methodology, statistical analysis, and data management: Sandrine Andrieu, Christelle Cantet, Christophe Morin; Multidomain group: Gabor Abellan Van Kan, Charlotte Dupuy, Yves Rolland (physical and nutritional components), Céline Caillaud, Pierre‐Jean Ousset (cognitive component), Françoise Lala (preventive consultation). The cognitive component was designed in collaboration with Sherry Willis from the University of Seattle, and Sylvie Belleville, Brigitte Gilbert, and Francine Fontaine from the University of Montreal.

Co‐Investigators in associated centers: Jean‐François Dartigues, Isabelle Marcet, Fleur Delva, Alexandra Foubert, Sandrine Cerda (Bordeaux); Marie‐Noëlle‐Cuffi, Corinne Costes (Castres); Olivier Rouaud, Patrick Manckoundia, Valérie Quipourt, Sophie Marilier, Evelyne Franon (Dijon); Lawrence Bories, Marie‐Laure Pader, Marie‐France Basset, Bruno Lapoujade, Valérie Faure, Michael Li Yung Tong, Christine Malick‐Loiseau, Evelyne Cazaban‐Campistron (Foix); Françoise Desclaux, Colette Blatge (Lavaur); Thierry Dantoine, Cécile Laubarie‐Mouret, Isabelle Saulnier, Jean‐Pierre Clément, Marie‐Agnès Picat, Laurence Bernard‐Bourzeix, Stéphanie Willebois, Iléana Désormais, Noëlle Cardinaud (Limoges); Marc Bonnefoy, Pierre Livet, Pascale Rebaudet, Claire Gédéon, Catherine Burdet, Flavien Terracol (Lyon); Alain Pesce, Stéphanie Roth, Sylvie Chaillou, Sandrine Louchart (Monaco); Kristelle Sudres, Nicolas Lebrun, Nadège Barro‐Belaygues (Montauban); Jacques Touchon, Karim Bennys, Audrey Gabelle, Aurélia Romano, Lynda Touati, Cécilia Marelli, Cécile Pays (Montpellier); Philippe Robert, Franck Le Duff, Claire Gervais, Sébastien Gonfrier (Nice); Yannick Gasnier and Serge Bordes, Danièle Begorre, Christian Carpuat, Khaled Khales, Jean‐François Lefebvre, Samira Misbah El Idrissi, Pierre Skolil, Jean‐Pierre Salles (Tarbes).

MRI group: Carole Dufouil (Bordeaux), Stéphane Lehéricy, Marie Chupin, Jean‐François Mangin, Ali Bouhayia (Paris); Michèle Allard (Bordeaux); Frédéric Ricolfi (Dijon); Dominique Dubois (Foix); Marie Paule Bonceour Martel (Limoges); François Cotton (Lyon); Alain Bonafé (Montpellier); Stéphane Chanalet (Nice); Françoise Hugon (Tarbes); Fabrice Bonneville, Christophe Cognard, François Chollet (Toulouse).

PET scans group: Pierre Payoux, Thierry Voisin, Julien Delrieu, Sophie Peiffer, Anne Hitzel, (Toulouse); Michèle Allard (Bordeaux); Michel Zanca (Montpellier); Jacques Monteil (Limoges); Jacques Darcourt (Nice).

Medico‐economics group: Laurent Molinier, Hélène Derumeaux, Nadège Costa (Toulouse).

Biological sample collection: Bertrand Perret, Claire Vinel, Sylvie Caspar‐Bauguil (Toulouse). Safety management: Pascale Olivier‐Abbal

IHU HealthAge Open Science Group—Nicola Coley, Sandrine Andrieu, Christelle Cantet, Sophie Guyonnet
