# Supplementary material for: Mediation of modifiable risk factors in two multidomain dementia prevention trials
Source: Alzheimers Dement. 2025 Jan 27;21(2):e14557. doi: 10.1002/alz.14557 (PMC11851128; doi:10.1002/alz.14557)
Supplement: Supplementary file 1 — Supporting Information [file ALZ-21-e14557-s002.docx]

**Appendix A: Measurement of mediators**

BMI

preDIVA: Weight and height were measured on standardized scales. BMI was calculated as weight (kg)/ height^2^ (m)

MAPT: Weight and height were measured on standardized scales. BMI was calculated as weight (kg)/ height^2^ (m)

Blood pressure

preDIVA: Blood pressure was measured twice sitting using a standardized OMRON measurement device. The average is taken for the current analyses.

MAPT: Blood pressure was measured once reclining.

Total cholesterol

preDIVA: Total cholesterol was measured in full blood.

MAPT: Total cholesterol was measured in full blood.

Physical activity

preDIVA: Self-reported physical activity was assessed using the LASA Physical Activity Questionnaire (LAPAQ) [1]

MAPT: Self-reported physical activity was assessed using the short version of the Minnesota Leisure Time Physical Activity Questionnaire [2]

1) Stel VS, Smit JH, Pluijm SM, Visser M, Deeg DJ, Lips P. Comparison of the LASA Physical Activity Questionnaire with a 7-day diary and pedometer. J Clin Epidemiol. 2004 Mar;57(3):252-8. doi: 10.1016/j.jclinepi.2003.07.008.

2) Fried LP, Tangen CM,Walston J, Newman AB, Hirsch C, Gottdiener J, et al. Frailty in older adults: evidence for a phenotype. J Gerontol Ser A Biol Sci Med Sci 2001;56:M146–56.

**Appendix B: Verification proportional hazards assumptions**

Intervention effect on dementia: assumption holds


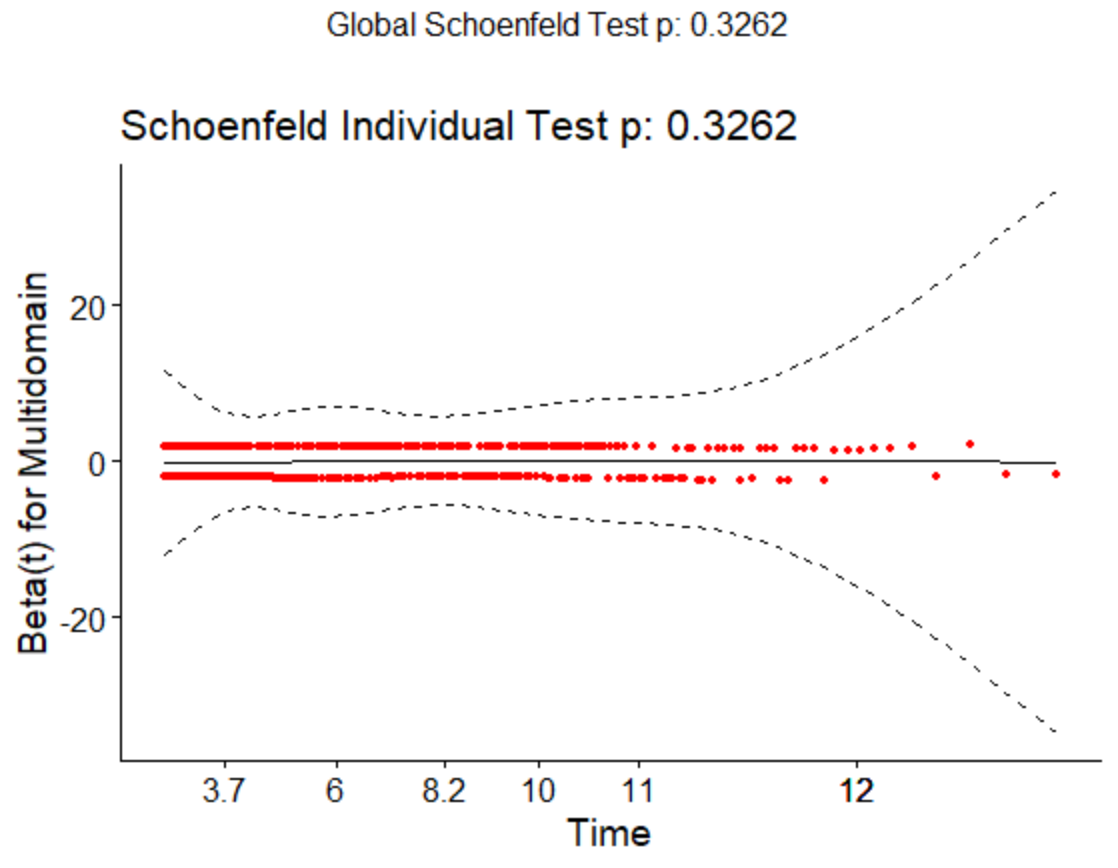


Change in risk factors in relation to dementia: assumption holds

Sidenote: The proportional hazard assumption did not hold for the association between total cholesterol and dementia incidence. There was no association between total cholesterol and dementia during the first eight years after start of the interventions, and after eight years the HR was 1.31 (1.00 to 1.72)
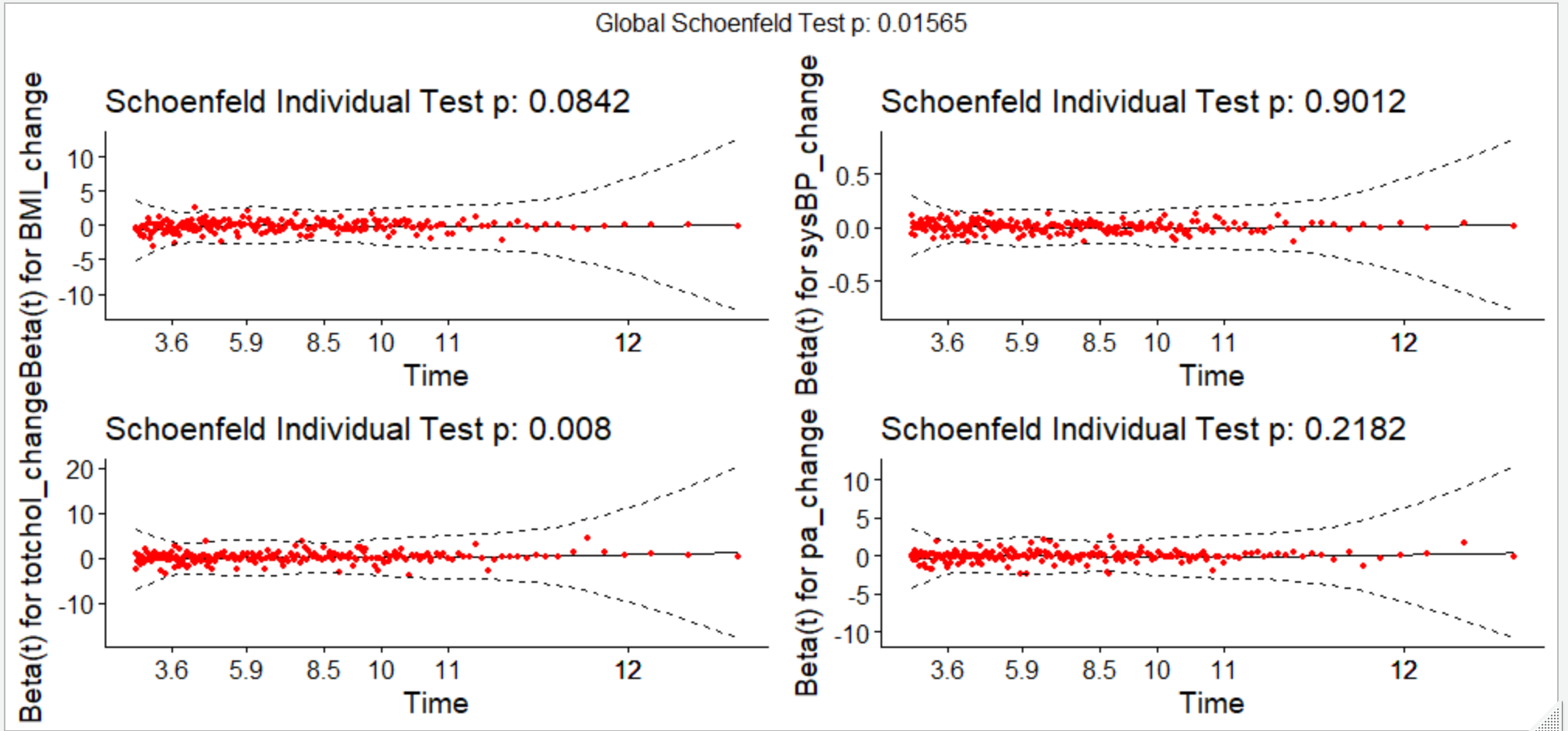


**Appendix C: R code of main MMA analysis**

x = DF %>% select(c('BMI_change', 'sysBP_change', 'totchol_change', 'pa_q_change', 'age', 'gender', 'educ', 'diab', 'cvd','Trial', 'BMI_BL', 'sysBP_BL', 'totchol_BL', 'pa_q_BL')) # all potential mediators and covarates

status <- DF$Dementia # outcome

y=Surv(DF$allcause_STY, DF$Dementia)

pred = DF %>% select(c('Multidomain')) # randomisation status

set.seed(523)

temp.cox.binx<-mma(x,y,pred=pred, contmed=c(1:4), alpha = 1, alpha2 = 1, n2 = 500)

summary(temp.cox.binx, plot = TRUE) # absolute mediation effects

**Appendix D: Baseline characteristics of those included and not included from the analyses**

| **Table D.1:** Participants’ characteristics for those included in the analyses and those not included in the analyses – due to missing data ^a^ | | |  |
| --- | --- | --- | --- |
|  | **Not included** | **Included** |  |
| N | 2310 | 2895 |  |
| Age, mean (SD)^b^ | 75.0 (3.3) | 74.6 (3.2) |  |
| Women, N(%) | 1302 (56.4%) | 1704 (58.9%) |  |
| Level of education, N(%)^b^ |  |  |  |
| Low | 435 (19.4%) | 486 (16.8%) |  |
| Medium | 1230 (54.9%) | 1587 (54.8%) |  |
| High | 574 (25.6%) | 822 (28.4%) |  |
| Cognition (MMSE), median [IQR]^b^ | 28 [27-29] | 29 [27-29] |  |
| Systolic blood pressure (mmHg) , mean (SD)^b^ | 151.5 (22.1) | 150.2 (21.7) |  |
| Total cholesterol (mmol/l) , mean (SD) | 5.3 (1.1) | 5.4 (1.1) |  |
| BMI (kg/m2) , mean (SD) | 27.1 (4.2) | 27.0 (4.2) |  |
| Moderate/vigorous activity (hr/wk) , mean (SD) | 7.3 (8.3) | 7.6 (8.6) |  |
| Diabetes, N(%) | 352 (15.3%) | 445 (15.4) |  |
| MI, N(%)^b^ | 572 (24.9%) | 637 (22.1) |  |
| Stroke, N(%) | 212 (9.3%) | 228 (7.9) |  |
| Incident dementia, N(%)^b^ | 249 (10.9) | 237 (8.2) |  |
| Incident alzheimer’s disease, N(%)^b^ | 134 (5.8) | 108 (3.7) |  |
| Cognition at year 4 (MMSE), median [IQR] | 29 [27-30] | 29 [28-30] |  |
| ^a^ Characteristics at baseline, unless otherwise indicated; ^b^ Significant at p <0.05; SD: Standard deviation, MMSE: Mini Mental State Examination, IQR: Inter Quartile Range, BMI: Body Mass Index, hr/wk: hours per week, MI: Myocardial Infarction | | |  |

**Appendix E: Mediation effects using a nonlinear approach (MART)**

| **Table E.1:** The mediating role of change in dementia risk factors in the effect of multidomain interventions on dementia incidence with the linear approach (main result) and the non-linear approach (MART) | | |  |
| --- | --- | --- | --- |
|  | Beta (95%CI) | HR (95%CI) | |
| **Dementia incidence (linear approach. main result)** |  |  | |
| Total effect | 0.07 (-0.29 to 0.45) | 1.07 (0.75 to 1.57) | |
| Direct effect | 0.09 (-0.25 to 0.48) | 1.08 (0.78 to 1.62) | |
| Indirect effect | -0.02 (-0.10 to 0.04) | 0.98 (0.90 to 1.04) | |
| Via BMI change | -0.00 (-0.05 to 0.05) | 1.00 (0.95 to 1.05) | |
| Via blood pressure change | -0.01 (-0.07 to 0.04) | 0.99 (0.93 to 1.04) | |
| Via cholesterol change | 0.00 (-0.01 to 0.02) | 1.00 (0.99 to 1.02) | |
| Via physical activity change | -0.01 (-0.04 to 0.00) | 0.99 (0.96 to 1.00) | |
| **Dementia incidence (non-linear approach, MART)** |  |  | |
| Total effect | -0.00 (-0.05 to 0.12) | 1.00 (0.95 to 1.13) | |
| Direct effect | 0.01 (-0.02 to 0.13) | 1.01 (0.98 to 1.14) | |
| Indirect effect | -0.01 (-0.03 to 0.02) | 0.99 (0.97 to 1.02) | |
| Via BMI change | -0.00 (-0.03 to 0.03) | 1.00 (0.97 to 1.03) | |
| Via blood pressure change | -0.01 (-0.03 to 0.00) | 0.99 (0.97 to 1.00) | |
| Via cholesterol change | 0.00 (-0.01 to 0.01) | 1.00 (0.99 to 1.01) | |
| Via physical activity change | -0.00 (-0.00 to 0.00) | 1.00 (1.00 to 1.00) | |
| Beta: for dementia absolute effects are mean log(HR)s of 500 bootstrap samples with bias-corrected and accelerated (BCa) bootstrap 95% confidence intervals between brackets. HR: Hazard ratio | | |  |
| All models are adjusted for trial, baseline mediator level, age, sex and education level, diabetes and cardiovascular disease. | | |  |

**Appendix F: Stepwise addition of potential confounders**

| **Table F.1:** Effects multidomain interventions on intermediates and outcomes | | | | | |
| --- | --- | --- | --- | --- | --- |
|  | **Crude model** | **Model 1** | **Model 2** | **Model 3** | **Model 4** |
| **Effects on intermediates^a^** |  |  |  |  |  |
| BMI change (MD, kg/m2) | 0.01 (-0.09 to 0.11) | 0.01(-0.10 to 0.11) | 0.01 (-0.10 to 0.11) | 0.03 (-0.07 to 0.13) | 0.02 (-0.08 to 0.13) |
| Blood pressure change (MD, mmHg) | -3.62 (-5.29 to -1.94) | -3.60 (-5.26 to -1.94) | -3.60 (-5.26 to -1.94) | -3.56 (-5.22 to -1.89) | -2.62 (-4.02 to -1.22) |
| Cholesterol change (MD, mmol/l) | -0.02 (-0.08 to 0.05) | -0.02 (-0.08 to 0.05) | -0.02 (-0.08 to 0.05) | -0.00 (-0.07 to 0.07) | -0.01 (-0.08 to 0.05) |
| Physical activity change (MD, quintile of time moderate/vigorous activity) | 0.10 (-0.03 to 0.22) | 0.09 (-0.02 to 0.22) | 0.09 (-0.03 to 0.22) | 0.09 (-0.03 to 0.22) | 0.10 (-0.02 to 0.21) |
| **Effects on outcomes** |  |  |  |  |  |
| Dementia incidence (HR) | 1.05 (0.81 to 1.36) | 1.07 (0.83 to 1.39) | 1.05 (0.81 to 1.37) | 1.08 (0.83 to 1.40) | 1.09 (0.84 to 1.41) |
| Cognition (MD, MMSE)^b^ | 0.03 (-0.13 to 0.17) | 0.03 (-0.11 to 0.17) | 0.04 (-0.10 to 0.18) | 0.05 (-0.09 to 0.19) | 0.05 (-0.09 to 0.19) |
| HR: Hazard Ratio, MD: mean difference between the intervention and control group, MMSE: Mini Mental State Examination at year 4.  All models have a random intercept for trial/centre/GP practice. Model 1: adjusted for age, sex and education level, model 2: additionally adjusted for diabetes and cardiovascular disease, model 3: additionally adjusted for change in (the other) intermediate factors, model 4: additionally adjusted for baseline levels of the intermediates  Effects on intermediates are analysed in dataset with complete data on dementia, results in dataset with complete data on cognition were similar  ^a^ Change between baseline and 2 years of follow up  ^b^ MMSE score at 4 years of follow up, all models additionally adjusted for baseline MMSE | | | | | |

| **Table F.2:** Associations intermediates with dementia incidence (HR) and cognition (MD) | | | | | |
| --- | --- | --- | --- | --- | --- |
|  | **Crude model** | **Model 1** | **Model 2** | **Model 3** | **Model 4** |
| **Dementia incidence (HR(95%CI))** |  |  |  |  |  |
| BMI change (per kg/m2) | 0.83 (0.76 to 0.92) | 0.84 (0.77 to 0.93) | 0.84 (0.77 to 0.93) | 0.84 (0.76 to 0.93) | 0.82 (0.74 to 0.91) |
| Blood pressure change (per 10 mmHg) | 1.02 (0.96 to 1.09) | 1.02 (0.96 to 1.09) | 1.02 (0.96 to 1.09) | 1.03 (0.96 to 1.09) | 1.04 (0.97 to 1.12) |
| Cholesterol change (per mmol/l) | 1.09 (0.94 to 1.26) | 1.10 (0.95 to 1.27) | 1.07 (0.92 to 1.24) | 1.06 (0.91 to 1.23) | 1.10 (0.93 to 1.29) |
| Physical activity change^c^ | 0.93 (0.85 to 1.01) | 0.94 (0.86 to 1.02) | 0.93 (0.86 to 1.02) | 0.94 (0.86 to 1.02) | 0.94 (0.85 to 1.04) |
| **Cognition^b^ (MMSE, MD (95%CI))** |  |  |  |  |  |
| BMI change (per kg/m2) | 0.07 (0.02 to 0.12) | 0.06 (0.01 to 0.11) | 0.06 (0.01 to 0.11) | 0.06 (0.01 to 0.11) | 0.06 (0.01 to 0.11) |
| Blood pressure change (per 10 mmHg) | 0.03 (0.00 to 0.07) | 0.03 (0.00 to 0.06) | 0.04 (0.00 to 0.07) | 0.03 (-0.00 to 0.06) | 0.02 (-0.02 to 0.06) |
| Cholesterol change (per mmol/l) | 0.04 (-0.04 to 0.11) | 0.03 (-0.04 to 0.10) | 0.03 (-0.04 to 0.11) | 0.03 (-0.05 to 0.10) | 0.02 (-0.06 to 0.10) |
| Physical activity change^c^ | -0.00 (-0.05 to 0.04) | -0.01 (-0.05 to 0.04) | -0.01 (-0.05 to 0.04) | -0.01 (-0.05 to 0.04) | 0.02 (-0.03 to 0.07) |
| HR: Hazard Ratio, MD: mean difference between the intervention and control group, MMSE: Mini Mental State Examination at year 4.  All models have a random intercept for trial/centre/GP practice. Model 1: adjusted for age, sex and education level, model 2: additionally adjusted for diabetes and cardiovascular disease, model 3: additionally adjusted for change in the other factors  ^b^ MMSE score at 4 years of follow up, all models additionally adjusted for baseline MMSE; ^c^ per quintile of time moderate/vigorous activity | | | | | |

**Appendix G: Mediation effects with Alzheimer’s disease as outcome**

| **Table G.1:** The mediating role of change in dementia risk factors in the effect of multidomain interventions on dementia incidence and Alzheimer’s disease | | |  |
| --- | --- | --- | --- |
|  | Beta (95%CI) | HR (95%CI) | |
| **Dementia incidence (main result)** |  |  | |
| Total effect | 0.07 (-0.29 to 0.45) | 1.07 (0.75 to 1.57) | |
| Direct effect | 0.09 (-0.25 to 0.48) | 1.08 (0.78 to 1.62) | |
| Indirect effect | -0.02 (-0.10 to 0.04) | 0.98 (0.90 to 1.04) | |
| Via BMI change | -0.00 (-0.05 to 0.05) | 1.00 (0.95 to 1.05) | |
| Via blood pressure change | -0.01 (-0.07 to 0.04) | 0.99 (0.93 to 1.04) | |
| Via cholesterol change | 0.00 (-0.01 to 0.02) | 1.00 (0.99 to 1.02) | |
| Via physical activity change | -0.01 (-0.04 to 0.00) | 0.99 (0.96 to 1.00) | |
| **Alzheimer’s disease** |  |  | |
| Total effect | 0.12 (-0.31 to 0.65) | 1.13 (0.73 to 1.92) | |
| Direct effect | 0.14 (-0.29 to 0.68) | 1.15 (0.75 to 1.97) | |
| Indirect effect | -0.02 (-0.15 to 0.06) | 0.98 (0.86 to 1.06) | |
| Via BMI change | -0.01 (-0.12 to 0.03) | 0.99 (0.89 to 1.03) | |
| Via blood pressure change | -0.02 (-0.13 to 0.02) | 0.98 (0.88 to 1.02) | |
| Via cholesterol change | 0.00 (-0.02 to 0.04) | 1.00 (0.98 to 1.04) | |
| Via physical activity change | -0.00 (-0.08 to 0.01) | 1.00 (0.92 to 1.01) | |
| Beta: for dementia absolute effects are mean log(HR)s of 500 bootstrap samples with bias-corrected and accelerated (BCa) bootstrap 95% confidence intervals between brackets. For cognition absolute effects are regression coefficients. HR: Hazard ratio | | |  |
| All models are adjusted for trial, baseline mediator level, age, sex and education level, diabetes and cardiovascular disease. | | |  |

**Appendix H: Mediation of smoking in effect intervention on dementia incidence**

Based on preDIVA data with observational extension for dementia

**Analyses**

Analyses are performed the same as the fully adjusted complete case model for the main analyses. All analyses were adjusted for baseline mediator, age, sex, education level, diabetes, cardiovascular disease, change in (the other) intermediate factors. Naïve analyses have a random intercept for centre/GP practice. MMA analyses are adjusted for trial.

**Results**

*Change in smoking in the first 2 years after randomization*

| **Table H** Change in smoking from baseline to year 2, (n (%)) | |
| --- | --- |
| Stable non-smoker | 1662 (87%) |
| Stable smoker | 181 (10%) |
| Quiter | 44 (2%) |
| Starter | 18 (1%) |

Effect intervention on dementia incidence: 1.08 (0.82 to 1.43)

Effect intervention on change in smoking (yes/no) (Mean difference): 0.004 (-0.012 to 0.021)

Association change in smoking on dementia incidence (Hazard ratio): 1.47 (0.62 to 3.52)

MMA analyses did not converge. Likely due to the very low number of people who changed smoking status.

**Conclusion**

Only 3% of preDIVA participants changed smoking status in the first 2 years. There was no mediation of smoking in the effect of the interventions on dementia incidence.

**Appendix I: Results non-mediation models using multiple imputation**

| **Table I.1:** Effects of multidomain interventions on intermediates and dementia | | | | | | | |  |  |
| --- | --- | --- | --- | --- | --- | --- | --- | --- | --- |
|  |  | | | **Complete case model** | **MI model** | |  |  |  |
| **Effects interventions on intermediates^a^** | | | | | | |  |  |  |
| BMI change (MD, kg/m2) |  | | | 0.02 (-0.08 to 0.13) | 0.01 (-0.10 to 0.11) | |  |  |  |
| Blood pressure change (MD, mmHg) |  | | | -2.62 (-4.02 to -1.22) | -3.61 (-5.10 to -2.11) | |  |  |  |
| Cholesterol change (MD, mmol/l) |  | | | -0.01 (-0.08 to 0.05) | 0.00 (-0.06 to 0.06) | |  |  |  |
| Physical activity change (MD, quintile of time moderate/vigorous activity) |  | | | 0.10 (-0.02 to 0.21) | 0.07 (-0.04 to 0.17) | |  |  |  |
| **Effects interventions on outcomes** |  | | |  |  | |  |  |  |
| Dementia incidence (HR) |  | | | 1.09 (0.84 to 1.41) | 0.98 (0.82 to 1.18)^b^ | |  |  |  |
| HR: Hazard Ratio, MD: mean difference between the intervention and control group, MI: Pooled analyses based on 15 imputed datasets. | | | | | | | |  |  |
| All models were adjusted for baseline mediator, age, sex, education level, diabetes, cardiovascular disease, change in (the other) intermediate factors and have a random intercept for trial/centre/GP practice. | | | | | | | |  |  |
| ^a^ Change between baseline and 2 years of follow up; ^b^ The MI model for the effects of the interventions on dementia incidence was adjusted for trial instead of the random intercepts due to incompatibility of COXME with the pool function in tydyR in R. | | | | | | | |  |  |
| **Table I.2:** Associations intermediates with dementia incidence (HR) | | | | | | | | |  |
|  | | |  | **Complete case model** | | | **MI model** | | |
| **Dementia incidence (HR(95%CI))** | | |  |  | | |  | | |
| BMI change (per kg/m2) | | |  | 0.82 (0.74 to 0.91) | | | 0.86 (0.80 to 0.93) | | |
| Blood pressure change (per 10 mmHg) | | |  | 1.04 (0.97 to 1.12) | | | 1.02 (0.96 to 1.08) | | |
| Cholesterol change (per mmol/l) | | |  | 1.10 (0.93 to 1.29) | | | 1.03 (0.88 to 1.19) | | |
| Physical activity change (per quintile of time moderate/vigorous activity) | | |  | 0.94 (0.85 to 1.04) | | | 0.95 (0.88 to 1.03) | | |
| HR: Hazard Ratio, MD: mean difference between the intervention and control group, MI: Pooled analyses based on 15 imputed datasets.  All models are adjusted for baseline mediator, age, sex, education level, diabetes, cardiovascular disease, change in (the other) intermediate factors. The complete case models have a random intercept for trial/centre/GP practice but the MI models were adjusted for trial instead of the random intercepts due to incompatibility of COXME with the pool function in tydyR in R. | | | | | | | | |  |
